# Optical Evaluation of New Designs of Multifocal Diffractive Corneal Inlays

**DOI:** 10.1155/2019/9382467

**Published:** 2019-11-11

**Authors:** Diego Montagud-Martínez, Vicente Ferrando, Juan A. Monsoriu, Walter D. Furlan

**Affiliations:** ^1^Centro de Tecnologías Físicas, Universitat Politècnica de València, Valencia 46022, Spain; ^2^Departamento de Óptica y Optometría y Ciencias de la Visión, Universitat de València, Burjassot 46100, Spain

## Abstract

**Purpose:**

To assess the imaging properties of two different designs of a new concept of corneal inlays whose working principle is based on diffraction.

**Methods:**

The quality of the retinal images provided by Diffractive Corneal Inlays (DCIs) was evaluated theoretically in comparison with Small Aperture Corneal Inlay (SACI). ZEMAX OpticStudio software was employed for the simulations in an eye model with different pupil diameters (3.0 mm and 4.5 mm). The employed merit functions in the analysis were the Modulation Transfer Function (MTF), the area under the MTF (MTFa), and the Point Spread Function (PSF). Comparison was made with the SACI at different defocus conditions.

**Results:**

The bifocal nature of the DCIs was demonstrated in a model eye for the first time. It was shown that the intensity of the near focus depends on the radius of the central zone. Retinal image quality of the DCI was equal to or exceeded the SACI in the majority of visual conditions as was demonstrated with simulated images.

**Conclusions:**

A new customizable type of corneal inlays has been evaluated using objective numerical simulations. Improvements in imaging of near objects and in light throughput compared with the popular small aperture inlays were demonstrated. These findings open a new technical branch of minimally invasive surgical solutions for the treatment of presbyopia.

## 1. Introduction

Presbyopia affects almost all adults over 45 years of age, and it has been estimated that globally there are more than 1.8 billion people with presbyopia, 820 million of whom had near visual impairment because they had no, or inadequate, vision correction [1].

At present, the minimum invasive surgical option for presbyopes who do not want glasses or contact lenses is to implant a corneal inlay. By means of a femtosecond laser, the surgeon creates a pocket inside the corneal stroma where the inlay is inserted, rendering the surgical procedure fast, simple, and importantly reversible.

Based on the working principle, different options have been launched in the market in the last years: corneal reshaping device, refractive corneal device, and small aperture corneal inlay (SACI) [2]. The last one, commercially known as Kamra® inlay (AcuFocus, Inc., Irvine, California, USA), is undoubtedly the most popular due to the reported good clinical outcomes [3, 4]. This device is an opaque disk of a biocompatible material (polyvinylidene fluoride) with a central aperture that produces an extended depth of focus. In addition, to facilitate the flow of nutrients to cells of the corneal stroma, the disk has a reduced external diameter and has more than 8,000 micropores, in a size range of 5–11 *μ*m diameter. Unfortunately, although SACI implantation can result in improved intermediate and near vision, it has several important intrinsic drawbacks. Firstly, only about twenty percent of the incident light passes through the disc's central aperture. Secondly, as much as five percent of incident light is diffracted by the disc's microholes. Thirdly, as the SACI is implanted monocularly, the interocular asymmetry induced by anisocoria combined with monovision deteriorates binocular summation [5] and stereoacuity [6].

In an effort to avoid these drawbacks, our group recently proposed a new concept of corneal inlays that take profit of the diffraction phenomena originated in the micropores of the SACI [7]. The result, DCI, is a device that, by exploiting the photon sieve concept [8], creates a diffractive focus for near vision in the implanted eye, on a personalized basis. In fact, an additional and important benefit of the DCI is that its optical characteristics (addition, intensity ratio between the near and far foci, and so on) can be modified by varying the size of the pinholes and the pattern of their distribution indicating that DCIs could be customized for a variety of specific patient's needs.

## 2. Materials and Methods

### 2.1. Model Eye

The assessment of the imaging properties of two different DCIs was investigated by implementing a schematic eye in the Zemax OpticStudio optical design software (http://www.zemax.com/os/opticstudio). The phakic model eye employed in the simulations was the Eye Retinal Image.zmx included in the Zemax software (see Table 1), in which the polychromatic receptor photopic spectral sensitivity is simulated using 470, 510, 555, 610, and 650 nm wavelengths, with relative weights, i.e., of 0.091, 0.503, 1.0, 0.503, and 0.107, respectively.

### 2.2. Corneal Inlays

**Figure 1:**
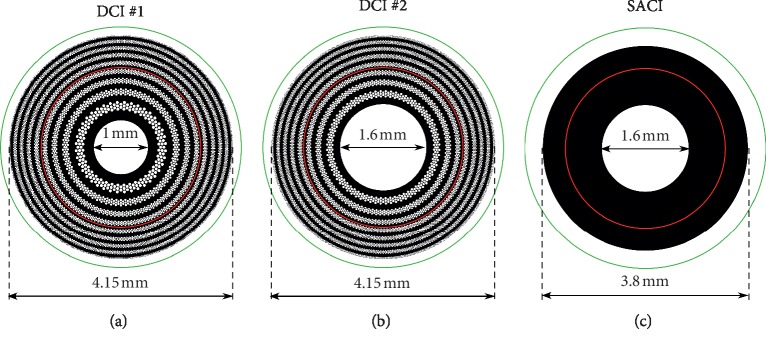
Diagrams of the corneal inlays evaluated in this study. The red and green circles represent 3.0 mm and 4.5 mm pupil diameters, respectively.

Two DCI models with an external diameter of 4.15 mm were evaluated in this study, both designed to provide a near focus corresponding to a typical addition of +2.50 D. Model DCI #1 was designed with a central hole of 1.00 mm diameter surrounded by 8 rings conformed by a total of 6394 holes. DCI #2 was designed with a central hole of 1.6 mm diameter surrounded by 8 rings with a total of 5989 holes. These two models have been considered to show the versatility in the DCI design and to study the influence on the resulting image performance of the central hole diameter. The external diameter corresponds to the original design [7]. A completely opaque SACI with the dimensions of the Kamra® has been evaluated in parallel as a reference. The inlays were located in the model eye at 0.20 mm from the anterior corneal surface as “User Defined Aperture” (uda) in ZEMAX, with the same radius of curvature and an asphericity of the anterior cornea surface (see Table 1). The inlay thickness was assumed as 5 *μ*m, and diagrams of the evaluated DCIs and SACI are shown in Figure 1.

### 2.3. Metrics

The image quality provided by the corneal inlays in this study was assessed using different merit functions. First, the MTFs were computed for different object vergences in the range from +0.5 D to –3.5 D in steps of 0.1 D. The best focus position of the retina remained the same for all MTF calculations. In each case, the MTFa was calculated as the numerical integral (using the trapezoid rule) for MTFs in a frequency range from 9.5 cpd to 59.9 cpd. These spatial frequencies correspond approximately to the sizes of letters of visual acuity charts between +0.5 logMAR and –0.2 logMAR.

Additionally, simulated images of a visual acuity test chart were obtained from the PSF provided by ZEMAX by means of the numerical convolution using a Matlab (MathWorks, Inc. R2018b) code. The simulations were performed with polychromatic light using 5 wavelengths as previously mentioned.

## 3. Results and Discussion

**Figure 2:**
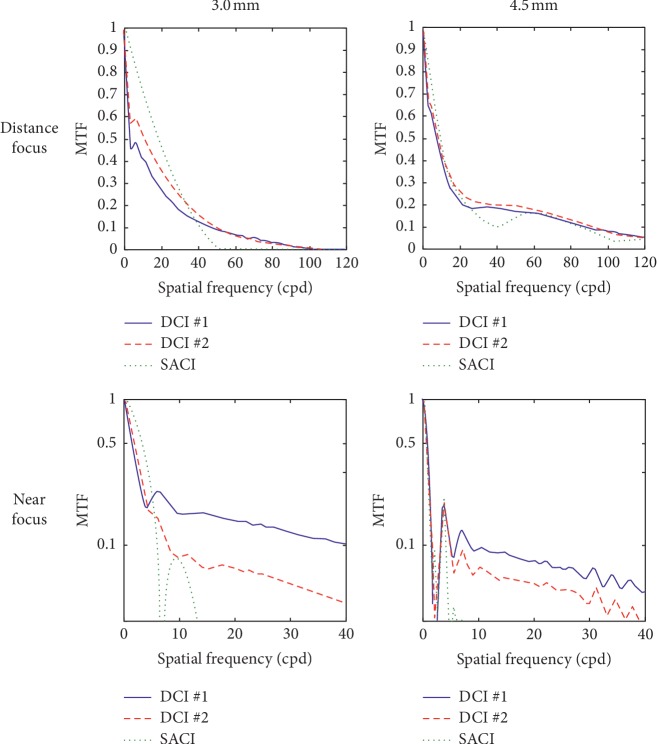
MTFs at the far and near foci provided by the three corneal inlays considered in this study.

Figure 2 shows the MTFs provided by the three corneal inlays at the far and near foci for 3.0 mm and 4.5 mm pupil diameters, simulating the eye response to photopic and mesopic lighting conditions, respectively. In order to enhance the differences, the MTFs in the near focus were represented on a logarithmic scale in the range from 0.03 to 1.

**Figure 3:**
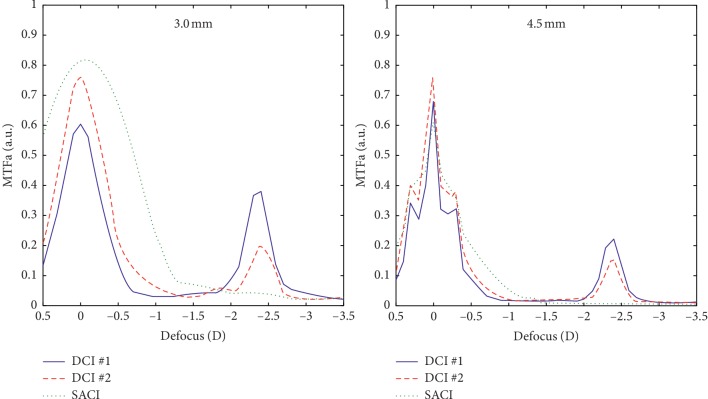
Comparative MTFa, in arbitrary units (a.u.), for 3.0 and 4.5 mm pupil diameters.

Note that, except for the distance focus and 3.0 mm pupil diameters, the performance of both models of DCI is superior to the SACI, even though the diffractive effects of the SACI (harmful for the image quality, in this case) have not been considered in the simulations. A better MTF curve was achieved by DCI #2 at the far focus for both pupils but with minimum differences. On the other hand, DCI #1 provides a better near focus than DCI #2. These results can also be verified in terms of area under MTF.  Figure 3 shows MTFa computed for 3.0 mm and 4.5 mm pupils in the range of frequencies that are important in terms of visual acuity.

For 3.0 mm pupil diameter, bigger differences can be observed between the three designs. DCI #1 has the lower values for the far focus, but the higher values for the near focus. These differences are attenuated for 4.5 mm pupil. In this case, all the three inlays have a comparable performance at the far focus, but both DCIs maintain an effective near focus.

**Figure 4:**
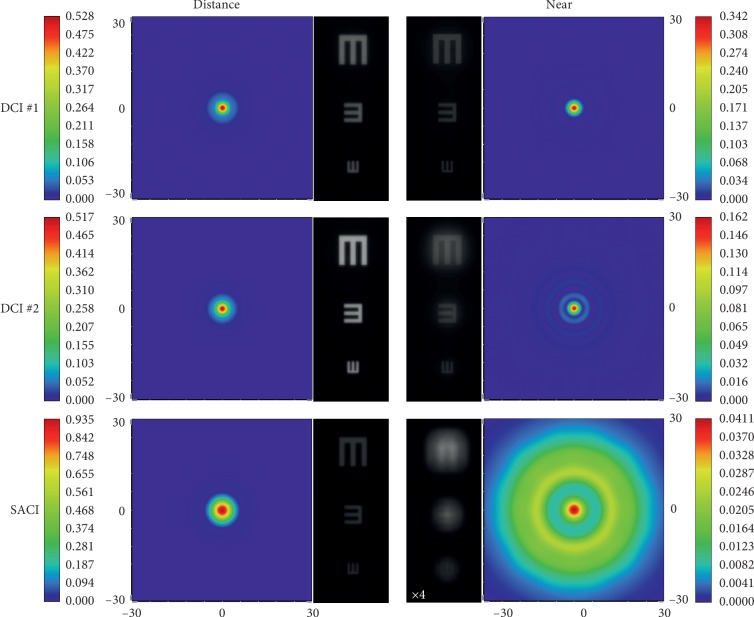
PSFs and the corresponding simulated images of the three inlays for distance and near objects Zemax model eye with a pupil diameter of 3.0 mm.

**Figure 5:**
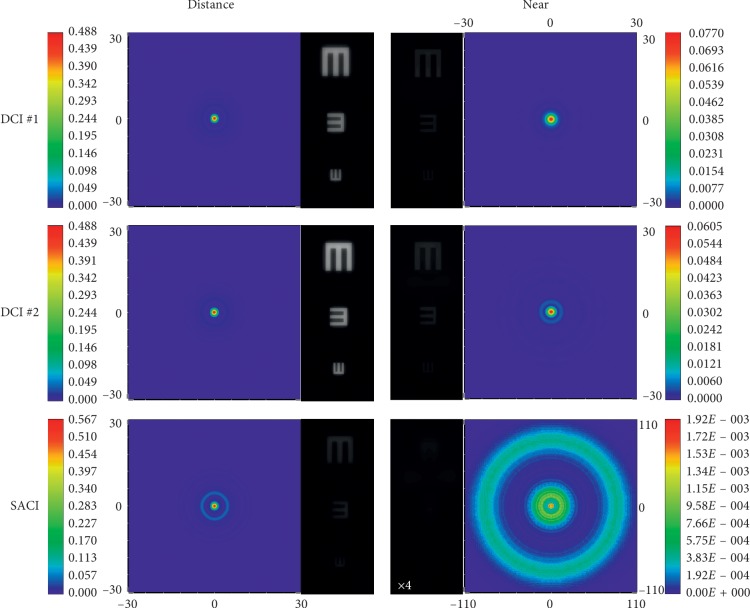
PSFs and the corresponding simulated images of the three inlays for distance and near objects Zemax model eye with a pupil diameter of 4.5 mm.

Figures 2 and 3 reveal the image quality of the studied corneal inlays; however, the main difference between the DCIs and SACI performance relies in the light throughput, which is more explicit in the comparison between the images obtained from the corresponding PSFs. Figures 4 and 5 show the PFSs provided by the model eye with two pupil diameters, virtually implanted with the different inlays, for point objects at far and near distances. Note that the scales of the PSFs are different, indicating the different intensities achieved with each inlay model. In these figures, the corresponding simulated images of three Snellen “E”s, with sizes corresponding to 0.4, 0.2, and 0 logMAR visual acuities are shown next to the corresponding PSF.

These images have been obtained as the convolution of the corresponding PSF with the test object. In this way, the relative intensity of the images and the spatial extension of the PSFs can be directly compared, except for the SACI at the near focus; in this case, the image intensity has been multiplied by a factor of 4 because otherwise this image would be almost black. Note that in Figure 5, the area of the PSF window has been extended to cover the spread of the PSF of the SACI at the near focus.

The image quality and the relative image intensity between them can be clearly observed in Figures 4 and 5. As can be seen, the image obtained with SACI is attenuated significantly. This is a very important fact because it was demonstrated that although the *binocular* distance visual acuity with a monocularly implanted SACI induces a *binocular* summation, the visual acuity for near distance seems to be close to the near distance acuity of the eye with SACI [9].

## 4. Conclusions

In conclusion, we performed an optical simulation on a new customizable treatment option for correcting presbyopia, the DCI. We found that the larger transmission of DCI compared with the SACI makes the proposed inlay highly luminous efficient, and its diffractive structure provides a near focus. Moreover, by using different models of the DCI, we have shown that the intensity ratio between the far and near foci can be controlled by adjusting the diffractive structure, which seems to be clinically relevant taking into account the particular patient's visual needs. In fact, in this study, we studied two different designs and demonstrated that the intensity of the foci of the DCIs depends on the radius of the central zone, being more intense in the near focus for the DCI #1 than for the DCI #2, but the opposite happens for the far focus. The PSFs and the simulated images show the improved performance of the DCI in comparison with the SACI, especially in near vision.

## Figures and Tables

**Table 1:** Phakic model eye with corneal inlay (CI).

Surface	Radius (mm)	Asphericity	Thickness (mm)	Refractive index
Anterior cornea	7.80	–0.50	0.20	1.377
Anterior CI	7.80	–0.50	0.005	1.377
Posterior CI	7.80	–0.50	0.315	1.377
Posterior cornea	6.70	–0.30	3.1	1.337
Iris	—	—	0.1	1.337
Anterior lens	10	0	3.7	1.42
Posterior lens	–6	–3.25	16.58	1.336

## Data Availability

The data supporting the results of the current article are available from the corresponding author upon request.
